# Driver self-regulation and depressive symptoms in cataract patients awaiting surgery: a cross-sectional study

**DOI:** 10.1186/1471-2415-13-45

**Published:** 2013-09-10

**Authors:** Michelle L Fraser, Lynn B Meuleners, Jonathon Q Ng, Nigel Morlet

**Affiliations:** 1 Curtin-Monash Accident Research Centre (C-MARC), School of Public Health, Curtin University, GPO Box U1987, Perth, WA 6845, Australia; 2 Eye & Vision Epidemiology Research Group, Perth, WA, Australia; 3 Centre for Health Services Research, School of Population Health, University of Western Australia, 35 Stirling Hwy, Crawley, WA 6009, Australia

**Keywords:** Older drivers, Cataract, Self-regulation, Depression

## Abstract

**Background:**

Cataract is an extremely common visual condition of ageing. Evidence suggests that visual impairment influences driving patterns and self-regulatory behavior among older drivers. However, little is known about the psychological effects of driver self-regulation among older drivers. Therefore, this study aimed to describe driver self-regulation practices among older bilateral cataract patients and to determine the association between self-regulation and depressive symptoms.

**Methods:**

Ninety-nine older drivers with bilateral cataract were assessed the week before first eye cataract surgery. Driver self-regulation was measured via the Driving Habits Questionnaire. Depressive symptoms were assessed using the 20-item Center for Epidemiological Studies Depression Scale. Visual, demographic and cognitive data were also collected. Differences between self-regulators and non self-regulators were described and linear regression modeling used to determine the association between driver self-regulation and depressive symptoms score.

**Results:**

Among cataract patients, 48% reported self-regulating their driving to avoid at least one challenging situation. The situations most commonly avoided were driving at night (40%), on the freeway (12%), in the rain (9%) and parallel parking (8%). Self-regulators had significantly poorer contrast sensitivity in their worse eye than non self-regulators (p = 0.027). Driver self-regulation was significantly associated with increased depressive symptoms after controlling for potential confounding factors (p = 0.002).

**Conclusions:**

Driver self-regulation was associated with increased depressive symptoms among cataract patients. Further research should investigate this association among the general older population. Self-regulation programs aimed at older drivers may need to incorporate mental health elements to counteract unintended psychological effects.

## Background

Cataract is an extremely common visual condition of ageing [1]. The number of older drivers on the roads with cataract is projected to increase dramatically over the coming decades. This is due to the ageing population and because older drivers are retaining their licenses for longer [2].

Cataract can negatively affect different aspects of vision including visual acuity, contrast sensitivity and stereopsis, potentially having serious consequences for driving ability [3]. Stereopsis, a form of depth perception, may be affected for bilateral cataract patients as it is influenced by binocular measures of visual acuity and contrast sensitivity as well as differences in vision between the two eyes [4]. Visual impairment due to cataract may lead to difficulty driving at night [5], in high traffic [5], recognizing signs, detecting hazards [6,7], judging vehicle speeds [8] and judging braking distances [9]. Previous research has also reported that older drivers with cataract are more likely to be involved in an at-fault crash [10] and have poorer driving performance [11] than those without cataract. Fortunately, surgery is a highly effective treatment for cataract. Those who have surgery through the public system however, typically wait long periods before surgery and are potentially at risk on the road during this time. Anecdotal evidence suggests that cataract patients may self-regulate their driving during the waiting period for first eye surgery, potentially reducing their risk of crashing.

Driver self-regulation involves the older individual evaluating their own functional abilities and making “adjustments in their driving behavior that adequately match their changing cognitive, sensory, and motor capacities” [12]. Driver self-regulation may include reducing total kilometers travelled, reducing distance travelled from home, driving only in familiar areas or reducing/ eliminating driving in challenging situations, such as at night [13].

Recently, there has been considerable interest in driver self-regulation as a potential road safety strategy for the ageing population. It should be noted that it has not been established whether older drivers are able to recognize their need for self-regulation and make changes that are appropriate for their actual driving ability, without resulting in dangerous on-road situations or over-restriction of driving [14]. In particular, those with cognitive impairment may lack the ability to appropriately self-regulate their driving [15]. Despite this, self-regulation has the potential to improve road safety by reducing exposure to driving conditions that older people find difficult, while allowing them to remain mobile and autonomous [13,14,16]. A number of education programs have also been developed in this area [17,18]. This may be because promoting self-regulatory practices represents a relatively cheap strategy compared to government intervention in older driver licensing [19].

Overall, self-regulation has been viewed as a positive adjustment and alternative to driving cessation, allowing older adults to drive safely for longer [20]. However, self-regulation still limits a person’s mobility including where and when they can drive [21]. It is well known that driving can provide older adults with mobility, social activity, independence, self-worth, control and can influence their health and well-being [22-24]. Not surprisingly, driving cessation has been shown to have a myriad of negative effects including reduced social functioning [25], social isolation [26] and increased depressive symptoms [24,27-29]. However, little is known about whether self-regulation is associated with negative psychological outcomes for older drivers.

Only one study to date has examined the association between self-regulation without cessation, and depressive symptoms among older drivers. The USA-based study involved drivers aged 70 years and older and used multinomial logistic regression to examine how driving reduction contributed to increases in depressive symptoms, measured using the abbreviated Center for Epidemiological Studies Depression Scale (CES-D) [27]. It was reported that those who reduced their driving distances were at increased risk of worsening depressive symptoms [27].

Evidence suggests that visual impairment influences driving patterns and self-regulatory behavior among older drivers [10,30,31]. In developed countries, visual impairment due to cataract is usually only temporary due to the effectiveness of surgery. Currently, little is known about whether cataract patients self-regulate their driving while waiting for surgery or whether self-regulation is associated with increased depressive symptoms, after controlling for potential confounding factors such as age among this group.

Therefore, this paper aims to describe driver self-regulation practices among older bilateral cataract patients while waiting for first eye surgery, and to determine the association between driver self-regulation and depressive symptoms.

## Methods

### Study design

A cross-sectional analysis was undertaken using data from a prospective study. Information was collected from older drivers with bilateral cataract, the week before first eye cataract surgery. Ethics Committee approval was obtained from the Curtin University Human Research Ethics Committee and the three Perth teaching hospitals involved. This study followed the Tenets of the Declaration of Helsinki.

### Participants

A total of 99 participants were recruited from three public hospitals in Perth, Western Australia between October 2009 and December 2010. All participants (aged 55 years or older) were drivers, had bilateral cataract and were scheduled to undergo first eye cataract surgery by phacoemulsification. Participants were excluded from the study if they had a confirmed diagnosis of Dementia, Parkinson’s Disease, had significant psychosis, were wheelchair bound, had other significant ocular conditions, were undergoing combined ocular surgery or did not speak English. Fourteen (14.1%) participants had co-morbid ocular conditions including glaucoma, macular degeneration and diabetic retinopathy. However, it was confirmed by an Ophthalmologist that the condition was either controlled or non-advanced and that cataract was the principal reason for vision loss. Eligible participants were recruited consecutively with a response rate of 93%.

### Data collection

Before any information was collected, informed written consent was obtained from each participant. Participation was entirely voluntary and patients were informed they could withdraw from the study at any time without consequence for their cataract treatment. Information on socio-demographic variables and health status were collected. Participants’ medical records were also reviewed to confirm co-morbid medical conditions and to obtain information on other ocular conditions.

Three vision-related tests were performed under standard conditions. Current correction was used for visual testing if participants wore their corrective lenses for daily activities. Visual acuity (surgery eye, non-surgery eye and binocular) was measured using an Early Treatment Diabetic Retinopathy Study (ETDRS) chart [32]. The chart was calibrated for a three meter distance and scored using a letter by letter method [32]. Scores were expressed on a logarithm of the minimum angle of resolution (logMAR) scale and those who could not read any letters were assigned a score of 1.3 logMAR units. Contrast sensitivity (surgery eye, non-surgery eye and binocular) was measured using a Pelli-Robson chart in log units [33]. The test was administered at a distance of one meter and scored using a letter by letter method [33]. Stereopsis was assessed by the Titmus Fly Stereotest (Stereo Optical Co., Inc.) which measured disparity from 1.602 to 3.447 log seconds of arc. Participants who could not see any images were assigned a score of 3.653 log seconds of arc. Cognitive ability was assessed using the Mini Mental State Examination (MMSE) [34]. This instrument contains questions relating to orientation to place, attention, calculation and recall. It can be used as a screening tool for cognitive impairment but cannot be used to diagnose dementia or other cognitive disorders. Responses on the test were totaled to produce a score between zero and 30 points, with higher scores representing better cognitive ability [34]. Since cognitive ability may affect driver self-regulation, the MMSE was used to control for cognitive ability in this study and was analyzed as a continuous variable.

Information on driver self-regulation behavior was collected via the driving difficulty section of the Driving Habits Questionnaire [10]. The self-reported driving difficulty scale contained eight items, each measured on a five point scale. These items related to driving in the rain, driving alone, parallel parking, making turns across traffic, driving on the freeway, in high traffic, at peak hour and at night. If participants responded that they had stopped driving in that situation due to their vision, they were considered to self-regulate on that item. If they responded otherwise or did not drive in that situation for reasons other than their vision, they were considered to not self-regulate on that item. Driver self-regulation before surgery was examined as a binary variable, with participants who self-regulated on one or more of the Driving Habits Questionnaire items considered self-regulators and those who self-regulated on no items, non self-regulators.

Depressive symptoms were measured using the 20-item Center for Epidemiological Studies Depression Scale (CES-D) [35]. A continuous overall score between zero and 60 was produced, with higher scores representing more depressive symptoms.

### Statistical analysis

Descriptive statistics were used to summarize all variables of interest. Independent samples t-tests and chi squared tests were used to compare self-regulators and non self-regulators. To determine the association between driver self-regulation status and depressive symptoms score, linear regression modeling was undertaken. The outcome of interest was the depressive symptoms score. Driver self-regulation status (yes or no) was entered as an explanatory variable. Potential confounding factors controlled for using regression modeling were sex, age, country of birth, living situation, education, medical conditions and cognition. Binocular measures of visual acuity and contrast sensitivity were highly correlated with visual acuity (r = 0.88, p < 0.001) and contrast sensitivity (r = 0.79, p < 0.001) in the better eye. The model was tested using either binocular measures or better/ worse eye measures. None of these visual measures were significantly related to depressive symptoms and it made little difference to the model whether binocular or better/worse eye measures were included. The model presented in this paper contains the visual variables: worse eye visual acuity, better eye visual acuity, worse eye contrast sensitivity, better eye contrast sensitivity and stereopsis. Explanatory variables with two sided p-values less than 0.05 were considered significant. All statistical analyses were performed using the SPSS package, version 18 (SPSS Inc, Chicago, USA).

## Results

The 99 bilateral cataract patients were aged 55 to 88 years with a mean of 72 years (SD: 7.9). Approximately half the participants were male (50.5%) and born in Australia (46.5%). Participants had an average of 3.1 chronic conditions (SD: 1.6), the most common being circulatory conditions (79.8%), musculoskeletal conditions (55.6%) and diabetes (31.3%). Fifty four participants (54.5%) were married and 37 (37.4%) lived alone. For the majority (72.7%), primary or secondary school was their highest level of education.

Almost half of participants (47.5%) reported they did not drive in at least one challenging situation, due to their visual impairment. These participants were considered to be self-regulators. Non self-regulators comprised 52.5% of the sample. The challenging driving situations most commonly avoided were driving at night (40.4%), on the freeway (12.1%), in the rain (9.1%) and parallel parking (8.1%). Only three percent of participants self-regulated their driving during peak hour traffic, and only one percent did not drive alone, make turns across traffic or drive in high traffic due to their vision.

Table 1 presents the demographic, health and visual characteristics of the sample by driver self-regulation status. Self-regulators had significantly poorer contrast sensitivity in their worse eye, seeing four letters less on average, than non self-regulators (p = 0.027). They also had significantly higher (worse) depressive symptoms scores than non self-regulators by 6.6 points (p = 0.001). There were no other significant differences between the two groups.

**Table 1:** Demographic, health and visual characteristics of bilateral cataract patients by driver self-regulation status (n = 99)

**Characteristic**	**Self-regulator**		**P value**
	**Yes (n = 47)**	**No (n = 52)**	
**Sex: n (%)**			
Male	22 (46.81%)	28 (53.85%)	
Female	25 (53.19%)	24 (46.15%)	0.484
**Age: mean (SD)**	71.40 (8.13)	72.60 (7.67)	0.455
**Country of birth: n (%)**			
Australia	22 (46.81%)	24 (46.15%)	
Not Australia	25 (53.19%)	28 (53.85%)	0.948
**Living situation: n (%)**			
Not alone	30 (63.83%)	32 (61.54%)	
Alone	17 (36.17%)	20 (38.46%)	0.814
**Education: n (%)**			
Primary or secondary school	34 (72.34%)	38 (73.08%)	
Higher education	13 (27.66%)	14 (26.92%)	0.935
**Cognition (MMSE score): mean (SD)**	27.43 (2.22)	27.38 (2.78)	0.931
**Number of chronic conditions: mean (SD)**	3.36 (1.54)	2.85 (1.59)	0.105
**Visual acuity (logMAR units): mean (SD)**			
Worse eye visual acuity ^a^	0.63 (0.35)	0.56 (0.25)	0.227
Better eye visual acuity ^a^	0.30 (0.18)	0.27 (0.13)	0.332
Bilateral visual acuity ^a^	0.25 (0.21)	0.21 (0.13)	0.204
**Contrast sensitivity (log units): mean (SD)**			
Worse eye contrast sensitivity	1.07 (0.48)	1.27 (0.35)	0.027*
Better eye contrast sensitivity	1.47 (0.20)	1.53 (0.16)	0.107
Bilateral contrast sensitivity	1.53 (0.21)	1.57 (0.17)	0.332
**Stereopsis **^a^**(log seconds of arc): mean (SD)**	2.34 (0.71)	2.11 (0.49)	0.068
**Depressive symptoms **^a^**(CES-D score): mean (SD)**	11.72 (9.50)	6.29 (5.10)	0.001*

*MMSE* Mini Mental State Examination, *CES-D* Center for Epidemiologic Studies Depression Scale, *logMAR* logarithm of the minimum angle of resolution, *SD* standard deviation.

* significant at p < 0.05.

^a^ Lower scores represent better scores.

Table 2 presents the results of the linear regression analysis examining the association between driver self-regulation and depressive symptoms score. After controlling for potential confounding factors and visual measures, self-regulation status was significantly associated with depressive symptoms. Cataract patients who self-regulated their driving had significantly higher (worse) depressive symptom scores than those who did not (p = 0.002). The only other factor significantly associated with depressive symptoms score was education level. Those who had completed higher education had significantly less depressive symptoms compared to those who had completed primary or secondary school only (p = 0.023). None of the visual measures were associated with depressive symptoms score in the linear regression model. It should also be noted that none of the visual measures were significantly associated with depressive symptoms score in univariate analyses. If the regression model was tested with the driver self-regulation status variable removed, there was still no significant association between the visual variables and depressive symptoms score. Interactions between the significant main effects were tested but were not significant.

**Table 2:** Factors associated with depressive symptoms score for bilateral cataract patients waiting for first eye surgery (n = 99)

**Variable**	**Coefficient**	**95% CI**		**P value**
Constant	30.85	−8.62	70.32	0.124
Driving self-regulator: yes	4.97	1.88	8.07	0.002*
Sex: Female	2.55	−0.83	5.93	0.138
Age (years)	−0.18	−0.40	0.05	0.122
Country of birth: Not Australia	−1.03	−4.02	1.96	0.494
Living situation: Not alone	0.70	−2.60	4.00	0.674
Education: Higher education	−4.14	−7.70	−0.58	0.023*
Number of chronic conditions	0.53	−0.49	1.54	0.308
Cognition (MMSE score)	−0.31	−1.02	0.40	0.385
Worse eye visual acuity	2.94	−7.20	13.07	0.566
Better eye visual acuity	−3.86	−15.05	7.33	0.495
Worse eye contrast sensitivity	−0.08	−7.17	7.01	0.983
Better eye contrast sensitivity	−1.10	−11.28	9.08	0.830
Stereopsis	−1.67	−6.15	2.81	0.460

*CI* confidence interval, *MMSE* Mini Mental State Examination.

* significant at p < 0.05.

## Discussion

This study found that a high proportion of bilateral cataract patients self-regulated their driving while waiting for cataract surgery. It also found that self-regulation was associated with increased depressive symptoms amongst this group, measured at one week prior to first eye cataract surgery.

Among cataract patients in this study, 48% reported self-regulating their driving to avoid at least one challenging driving situation. In contrast, two previous studies that used a modified version of the DHQ, found low levels of avoidance of challenging driving situations in the general older population [14,36]. The most commonly avoided driving situations among cataract patients were driving at night, on the freeway, in the rain and parallel parking. These challenging driving situations have also been found to be the most commonly avoided among the general older population [14,36].

Past research has indicated that older age and being female are associated with higher levels of driver self-regulation among older drivers [12,30,31]. For cataract patients, neither of these factors were significantly associated with driver self-regulation, however, this may be due to the small sample size of this study. Those who self-regulated their driving had significantly poorer contrast sensitivity (ability to detect differences in contrast between an object and its background) in their worse eye, than non self-regulators. The contrast sensitivity measure has also been previously identified as an important predictor of stopping or reducing driving among older drivers in general. [30,31]. However, these studies reported an association between binocular [30] or better eye [31] contrast sensitivity and driving cessation/ reduction. Among cataract patients, Owsley et al. reported that contrast sensitivity was the only independent predictor of crash involvement in the previous five years [37]. They also found that the relationship was stronger for worse eye contrast sensitivity than better eye [37]. While current evidence suggests that contrast sensitivity may influence driver self-regulation, as well as other driving outcomes, further research is required to understand the relationship with the worse eye, better eye and binocular measures.

This is one of the first studies to report that driver self-regulation was significantly associated with increased depressive symptoms, even after controlling for potential confounding factors and vision. This suggests that involuntary restriction of driving for reasons such as cataract, even temporarily, may impact on the psychological health of older adults. It is well known that many older drivers change or restrict their driving patterns due to lifestyle choice or convenience [12,19,27] and it is unlikely that such changes would have any negative psychological effects [24]. A longitudinal study by Windsor et al. found that “perceived control” was an important mediator of the association between driving cessation and depressive symptoms among older adults [24]. Since drivers in this study were self-regulating as a direct result of their visual impairment, rather than voluntarily, “perceived control” may help to explain the findings.

It is not possible however, to accurately determine the direction of the relationship between driver self-regulation and depressive symptoms using a cross-sectional study design. While it has been reported that depressive symptoms may increase the likelihood of restricting or stopping driving among older adults [30,31], a large USA-based cohort study investigated the direction of the relationship and found that driving cessation affected depression and not vice versa [29]. Another cohort study also found that driving cessation and restriction of driving distance affected depressive symptoms [27].

Interestingly, none of the visual measures were associated with depressive symptoms in the current study. While few studies have examined the association between visual measures and depression among cataract patients, poorer binocular visual acuity was found to be associated with depressive symptoms, measured using the Hospital Anxiety and Depression Scale, among elderly women with bilateral cataract [38]. Change in depressive symptoms after surgery was not found to be associated with changes in any visual measures [38]. It is important for larger longitudinal studies to examine whether deterioration of particular visual measures while waiting for first eye cataract surgery is associated with increased depressive symptoms. Such information would allow cataract patients at risk of depression to be identified and prioritized for surgery.

There were several limitations to this study. The cross-sectional design of the study could not determine cause and effect between driver self-regulation and depressive symptoms or examine how changes in self-regulation relate to depressive symptoms over time. There was also no comparison group of non-drivers with similar visual impairment to compare depressive symptoms to. In addition, we were unable to assess other visual measures including visual field in this study. Collecting variables including time spent on the cataract surgery waiting list and cataract severity may be useful in future studies of depressive symptoms among cataract patients. The Driving Habits Questionnaire was also self-reported making it potentially vulnerable to biases including recall and social desirability bias. To date, the Driving Habits Questionnaire has not been validated against actual self-regulation behaviors. The measure of self-regulation in this study was also restricted to avoidance of eight driving tasks which may not have covered all situations that drivers avoid [36] and did not include restriction of distances travelled.

Despite these limitations, this study controlled for a wide range of potential confounding factors and provided initial evidence that there may be an association between driver self-regulation and increased depressive symptoms among older adults waiting for cataract surgery. It is recommended that longitudinal studies with larger sample sizes be undertaken to determine whether driver self-regulation may increase depressive symptoms in the general older population. Driver self-regulation is a favorable alternative to driving cessation for the ageing population. However, if road safety strategies intend on using self-regulation education programs to improve older driver safety in the future, it may be necessary to incorporate mental health elements to counteract any unintended psychological effects.

## Conclusions

This study found that driver self-regulation was associated with increased depressive symptoms among cataract patients. Further research should investigate this association among the general older population. Self-regulation programs aimed at older drivers may need to incorporate mental health elements to counteract unintended psychological effects.

## Competing interests

The authors declare that they have no competing interests.

## Authors’ contributions

MF planned the study, conducted the data analysis and wrote the paper. LM, JN and NM contributed to the planning of the study and revising of the paper. All authors read and approved the final manuscript.

## Pre-publication history

The pre-publication history for this paper can be accessed here:

http://www.biomedcentral.com/1471-2415/13/45/prepub
